# Latino Family Childcare Providers' Beliefs, Attitudes, and Practices Related to Promotion of Healthy Behaviors among Preschool Children: A Qualitative Study

**DOI:** 10.1155/2015/409742

**Published:** 2015-03-19

**Authors:** Ana C. Lindsay, Judith A. Salkeld, Mary L. Greaney, Faith D. Sands

**Affiliations:** ^1^Department of Exercise and Health Sciences, University of Massachusetts Boston, 100 Morrissey Boulevard, Boston, MA 02125, USA; ^2^Harvard School of Public Health, Boston, MA 02115, USA; ^3^University of Rhode Island, Kingston, RI 02881, USA

## Abstract

*Background*. The continuing rise of obesity among Latinos is a public health concern with an immediate need for early prevention. Changes in family structures have increased demand and reliance for child care for young children. Latino children are the fastest-growing segment of the child population in the United States, and research shows that Latino families use preschools and day care centers much less than those of other ethnic groups, apparently because of cultural preferences for family-like care. *Objectives*. Given that many low income Latino children attend family child care homes (FCCHs), there is a need to explore the role that FCCH providers may play in establishing and reinforcing children's early healthful eating and physical activity behaviors and consequently in the prevention of childhood obesity. *Design*. Using purposive sampling, six focus groups were conducted in Spanish with licensed Latino FCCH providers (*n* = 44). Data was analyzed to identify recurrent themes. *Results*. Latino FCCH providers described how they play an influential role in promoting healthful eating and physical activity behaviors of preschool children in their care. They also identified many barriers and challenges in establishing and maintaining healthful nutrition and physical activity behaviors, including high cost of healthy foods, cold weather, and physical environment of FCCH. *Conclusions*. Latino FCCH providers can have a strong impact in promoting healthful behaviors in low-income, Latino communities. They may be able to effectively deliver interventions targeting low-income, minority families to promote healthful eating and physical activity behaviors and prevent child obesity.

## 1. Introduction

Latinos are the largest and most rapidly growing population group in the United States. Although rates of childhood obesity are still high for the general child population, children of low-income, minority families are at a particularly high risk of overweight and obesity [1]. The continuing rise of obesity within minority and immigrant populations, particularly Latinos, remains a pressing public health concern with an immediate need for early prevention.

Children living in the United States live in a society that has changed dramatically since the obesity epidemic first developed. Changes in family structure, gender roles, and families' needs for economic security have increased the demand and reliance on child care for children at increasingly younger ages. In addition, welfare reform laws requiring employment have increased the number of employed low-income parents and have contributed to an increase in the number of children enrolled in child care programs [2]. In 2012, 68% of women with children under the age of 6 were either working or looking for work, and almost 11 million preschool-aged children received some form of child care while their mothers worked [2, 3].

The child care setting is an important social environment that potentially influences the development of children's early dietary and physical activity habits and consequently contributes to the development of child overweight [4–6]. Given parents increasing reliance on child care settings for their children at continually younger ages, these settings are likely important venues for the implementation of programs and policies to help children develop healthful eating and physical activity habits [2, 4].

Child care providers, like parents, help establish and reinforce early healthful eating and physical activity habits among young children, and can be key players in preventing childhood obesity by developing a child care environment that fosters healthful eating and physical activity behaviors among children [7–9]. Child care provider's knowledge of nutrition and physical activity, the selection of food and meals, structure within their day care, and their own modeling of behaviors are all influential in young children's development of lifelong habits that contribute to normal weight or to overweight and obesity [10–12]. In fact, research suggests that child care providers may be more influential than or equally as important as parents in shaping food preferences of young children [13, 14]. Child care settings may help establish and reinforce children's eating and physical activity habits [4, 14, 15]. Furthermore, studies have found that Latino parents who send their children to child care believe these settings are instrumental in shaping and reinforcing the eating and physical activity of their children [14]. However, despite the growing number of studies and interventions targeting child care settings and given that more than 1.6 million children attend FCCHs [16], there is limited research examining FCCHs and their influence on the development of healthful eating and physical activity habits in Latino preschool-aged children, a group at increased risk of obesity [17].

In Massachusetts, the setting for this study, the majority of children enrolled in FCCHs are from minority backgrounds, including a high percentage of Latino children [18], and most Latino children attend FCCHs operated by Latino staff [18]. FCCHs are licensed by the Massachusetts Department of Early Education and Care (MA-EEC). FCCH providers are required to (1) have a plan for communicating with parents/guardians through various communication channels (e.g., handbooks, newsletters, and notes); (2) provide opportunities for outdoor and indoor active play; (3) have adequate indoor and outdoor space and equipment for active and safe play; and (4) provide opportunities for children to develop gross and fine motor skills [19]. State regulations do not specify the amount, frequency, and type of physical activity or regulate television use [19]. Additionally, FCCH providers may be eligible to participate in the Child and Adult Care Food Program (CACFP), which requires that CACFP participants follow USDA/CACFP guidelines for menus and feeding practices [20]. Given the growing importance of FCCHs in serving a large proportion of minority, low-income children, further research is needed to examine the role that FCCH providers play in establishing and reinforcing children's early healthful eating and physical activity habits and consequently preventing childhood obesity. Moreover, given the high rates of obesity among Latino preschoolers [17], and the fact that Latino families use preschools and day care centers much less than those of other ethnic groups, apparently because of cultural preferences for family-like care, there is a need to understand how family child care settings influence the development of eating, physical activity and sedentary behaviors associated with childhood obesity.

*“Preventing Obesity in Latino Family Child Care Homes”* is a multicomponent study employing qualitative methods to explore influences on eating habits, physical activity, sedentary behaviors, and ultimately risk of obesity among Latino preschool-aged children attending FCCHs in Massachusetts. Additionally, the study assessed practices, policies, and regulations of FCCHs that may be associated with risk of childhood obesity among Latino preschool-aged children. This current paper focuses on the results of the qualitative research examining Latino FCCH providers' beliefs and practices related to nutrition and feeding, and physical activity and sedentary behaviors among low-income preschool children.

## 2. Methods

### 2.1. Sample Selection and Recruitment

We worked with MA Department of Early Education and Care (EEC), which develops licensing regulations and requirements for childcare providers and supports training for early educators, and the Child Care Circuit, a nonprofit organization providing child care referrals, training, and parent and provider services, to identify cities in four regions of the state (North Shore, Greater Boston, Central MA, and Western MA) that have a large number of FCCHs run by Latino providers. MA-ECC and the Child Care Circuit, then identified two agencies that work directly with FCCH providers, CACFP, and Family Child Care Systems who compiled a list of all currently licensed Latino FCCH providers. From this list, we randomly selected 22 names per region of the state, with a goal of recruiting 8–12 individuals to participate in one focus group session in each region. We mailed all selected providers a recruitment flyer in Spanish that included a phone number that interested providers could call to obtain more information and/or express interest in participation. Interested providers who called spoke with a native Spanish speaker who explained the study and its purpose. A confirmatory/reminder phone call was made one to two days before the scheduled focus group session.

### 2.2. Focus Group Procedures

Focus group discussions were held in meeting rooms of public libraries, and all participants provided written informed consent. A native Spanish speaker trained in qualitative research methods conducted all focus groups in Spanish using a semistructured discussion guide including open-ended questions and probes. Focus group discussions were audio-taped with oral consent of participants. At completion of the focus group, participants received a $25 cash incentive and completed a brief demographic survey. The study protocol was approved by the Internal Review Board at the Harvard School of Public Health.

The focus group guide explored (1) providers' perceptions, attitudes and practices related to nutrition, and physical activity and sedentary behaviors; (2) influences of FCCH characteristics on children's eating and physical activity behaviors; (3) FCCH providers practices related to nutrition and physical activity; (4) educational activities offered by state and local agencies related to nutrition and physical activity and sedentary behaviors; (5) communication between FCCH providers and parents about FCCH practices and policies related to nutrition and physical activity and sedentary behaviors; and (6) barriers FCCH providers face in providing an environment conducive to healthful eating and physical activity behaviors. See the following for sample of questions used in focus group discussions with Latino family child care providers (FCCPs).

*FCCHs' Beliefs, Attitudes, and Practices Related to Child Feeding Practices*

- Please describe your routine, or plan, for meals that you give the children. (e.g., describe how you use menus, what times you serve meals?)
   Please tell me about your plan or routine for giving snacks to the children.
     For example, frequency that you give snacks to children, and so forth.
- What do you use to help you plan meals and snacks?
   For example, books, websites, agencies, other providers, and so forth.
- What things affect your choice of foods that you typically serve? Please give a few of the most important reasons for your food choices.
   For example, cost, rules, or guidelines from food program, cultural values and tradition, good for health, family member's advice, easy to prepare, and so forth.
- How much food you think a child should typically eat at a meal? (e.g.,* what is too much, not enough)*
- What are you and the children doing during meals? (e.g., sitting together at a table, watching TV or videos during meal)

*FCCPs' Beliefs, Attitudes, and Practices Related to Physical Activity*

- What are your ideas and thoughts about physical activity for children (in general)?
- Tell me about the routine you have for children to get physical activity while they are at the daycare.
   
*For example, how much time for PA daily, time of day, and so forth? *
- Why do you have this routine?
- What kinds of ways are children active outdoors on a typical day?
   
*For example, do they play games and use equipment?*
- What kinds of ways are children active indoors on a typical day?
   
*For example, do they play games, use equipment, and use videos or TV?*
- If you wanted to get more information about how much PA children need or get ideas about how to help kids be active, how would you do this?

*FCCPs' Beliefs, Attitudes, and Practices Related to Sedentary Behaviors.* We know that all children like to watch cartoons and other shows like Dora the Explorer. They also enjoy playing on a computer, cell phone, DS toys, and video and Nintendo games. We call these “screen time.”

- What are your thoughts in general on screen time for children aged 2 to 5?
- What rules or routines do you have about screen time?
- What do parents think about these rules?

*Providers' Beliefs, Attitudes, and Practices Related to Communication with Parents*

- How do you typically let parents know what their child did while at care, especially what they had to eat and what they did for physical activity?
- What, if anything, do you discuss with parents about their child's weight?
- Who would you talk to for advice if you thought a child was too thin or weighed too much? (family member, doctor, WIC staff, other)

### 2.3. Analysis

Audiotaped discussions were transcribed in Spanish and then translated into English by a bilingual consultant. The analysis plan, which used a content analysis approach, included an initial review of all translated transcripts by two members of the study team who also developed a codebook. Two coders trained in qualitative methods independently read and analyzed transcripts to identify salient convergent themes [21]. All transcripts were then coded based on broad categories of the areas of inquiry of the focus group guide. Inconsistencies in coding were discussed and resolved. Within these areas, emerging subthemes were identified and each one was assigned a specific code. Descriptive analyses and frequencies were calculated from sociodemographic questionnaires using Microsoft Excel 2008.

## 3. Results

In total, 44 providers (41 females, 3 males), all of whom are self-identified as Hispanic/Latino, participated in six focus groups. About one-third of participants (*n* = 14) had graduated from high school or earned their GED, and close to forty percent (*n* = 17) had attended some college. Most (*n* = 41, 93%) had several to up to 25 years of experience running FCCHs. Data analysis identified key themes related to nutrition and feeding practices, physical activity, and sedentary behaviors at Latino FCCHs. Emergent themes are discussed below, with quotes used to illustrate central points.

### 3.1. Nutrition and Food Practices

#### 3.1.1. Foods Served and Portion Sizes

Providers reported that foods served for breakfast often include 1% milk, fruit, yogurt, cereal, oatmeal, pancakes, and freshly squeezed or 100% juice. Lunch typically includes rice and/or beans, meat, and vegetables. Providers also mentioned that they do not allow or serve certain foods. Prohibited foods included juices that are not 100% juice, soda, hot dogs, and fried foods.

Providers indicated that they use guides from food programs (e.g., USDA) to determine portion sizes. Some mentioned using measuring cups to determine portion sizes while others spoke of using informal tools such as small plates. Many providers mentioned basing portion sizes on children's ages, which they believed to be an important factor in determining the quantity of food a child should eat.

Providers believed that children eat a healthier diet in their FCCHs than they do at home and that parents are supportive of FCCH policies.* “The parents that I have always feel good about what I give the kids because sometimes they do not have the time to prepare food like we prepare it with all of the nutrients, like vegetables, everything a child needs in a day.”* Most providers perceived parents as being too tired and busy to make healthy meals at home and that parents were more permissive then they were of their children eating unhealthy foods. “*Sometimes, if parents are alone, they do not make healthy meals because they do not have the motivation. They get home and ate too tired…”*

#### 3.1.2. Providers' Beliefs and Practices Related to Child Feeding

Most providers felt their role was to nurture and educate children in their care and viewed providing “good nutrition” and “healthy diets” as a priority. Several providers spoke of the need to compensate for unhealthy practices at home by parents. They also felt that it is important to expose children in their care to healthy foods and eating habits. Most providers reported being confident about their abilities to serve healthful foods at their FCCH and viewed themselves as “educators,” with the knowledge needed to teach children and their families about healthful diets.* “I try to inform the parents if there are activities in the community geared towards healthy eating and living for families … I give them pamphlets and all sorts of things that I have access to because of my work as a FCCH provider and that they may not be aware of.”*

In addition, most providers viewed themselves as “educators” and “professionals” and were vocal about wanting to discourage the perception of FCCHs providers as being just “*babysitters.*” Providers also spoke of enjoying seeing the children that they once cared for progressing, growing up, and being successful.* “It makes us feel good that we did the job we needed and those children will not have a hard time when they start school.”*

#### 3.1.3. Strategies to Incorporate Nutritious Foods

Providers across all focus groups spoke of multiple strategies they use to incorporate nutritious foods into meals and snacks. Strategies included becoming familiar with foods served and meal time practices at the homes of children attending their FCCH, introducing and encouraging new and healthy foods, and modeling of healthful behaviors. Some providers spoke of encouraging new foods by directly involving children with healthy food choices, as they believe this gives children a sense of control and increased openness to new foods, especially if they see their peers enjoying foods they would not normally eat.* “I usually allow children to help pick their foods as part of a game at the beginning of the week, so that they have some choice of preferred foods.” *In addition, some providers mentioned that children themselves influence food choices and eating habits of other children.* “At first a child may not want to eat something, but when they see another child eating it, they will try.”*

#### 3.1.4. Meal Planning

The majority of providers mentioned the importance of planning, buying, and preparing meals in advance. They felt planning ahead enabled them to serve a healthy and varied food menu on a weekly basis. Planning was seen as especially important for providers who served multiple meals (e.g., breakfast, a morning snack, lunch, an afternoon snack, and, in some cases, dinner).* “I like to plan ahead and know what I will serve the kids for at least a week … it's just much easier that way.”*

Across all focus groups, nearly all providers mentioned using available educational resources, including* Minute Menu* for their food shopping and planning needs of the week.* Minute Menu* is a computer software program affiliated with CACFP. A few providers mentioned that* Minute Menu* made it easy to “print a shopping list” for their meals and allowed for some flexibility in their preplanned menus.* “If a child does not like one vegetable they can substitute another vegetable in its place.”* Other resources included online “school-based menus” and menus provided by their local food programs such as* “Yours for Children.” *A few providers also reported that they use pamphlets from the USDA for snack and food ideas.* “They are always sending magazines on how to use the things to feed the children better and won't lead them into becoming obese children.”*

#### 3.1.5. Educational Workshops

The majority of providers mentioned participating in workshops about nutritional guidelines and using workshop resources to guide healthy eating options at their FCCHs. A few stated that attending these workshops and trainings caused them to make changes to their feeding practices and meal options at their FCCHs.* “I went to a workshop where they showed you how much sugar is in juice as measured by the number of sugar packets. After that workshop, I have just paid a lot more attention to serving juices to the children.”*

### 3.2. Factors That Influence Foods Served by FCCH Providers

#### 3.2.1. Cost of Healthy Foods

Several barriers to providing healthy foods in FCCHs were mentioned, including the high cost of healthy foods, especially organic and fresh fruits and vegetables.* “Something that affects is money, how much they [food program] pay you. Everything is so expensive, especially organic food. You have to pick out what is cheaper that week, fruit or vegetables, to be able to save money.”*

#### 3.2.2. Latino Culture

Most providers acknowledged that their Latino culture influences the foods they serve at their FCCH as well as eating routines and the foods that children eat with their families outside of the FCCHs.* “Cultures can positively or negatively affect people's food choices. A child comes to my home as an infant and grows with me. It does not matter what nationality they are. They learn to eat in my daycare and learn to eat food from my culture.”*

Although providers felt their Latino culture influences the foods they serve, they did not see their culture as negatively impacting foods they provided at their FCCHs. On the contrary, several providers noted several healthy food options that are part of the Latino culture, such as beans.* “I think that the Latino culture includes many food options. For example, I serve bean soup to the kids on a regular basis and that's very healthy for them.”*

#### 3.2.3. Perceptions of Child Weight Status

A few providers reported having some children “at risk” of overweight or obesity in their care and that this influenced their feeding practices, especially in determining portion sizes.* “I try to reduce the portion of the one that likes to eat so he won't get to eat too much and later be affected by obesity.” *While another added,* “I may have the child wait to see if they are still hungry then “give water or fruit” if the child is still hungry.”* Furthermore, some FCCPs felt that they needed to “control” what and how much children eat.* “We cannot let them eat everything they want.”*

#### 3.2.4. Perceptions of EEC and CACFP Policies

The majority of providers was aware and supportive of EEC and CACFP policies, regulations, and guidelines for both nutrition and physical activity behaviors of children attending FCCHs. They felt these policies made a real difference in the health of children attending FCCH.* “I think the EEC regulations require that children engage in an hour or more a day of PA, but a minimum of an hour a day. I agree with that.”*

### 3.3. Physical Activity and Sedentary Behaviors

#### 3.3.1. Beliefs about Physical Activity and Sedentary Behaviors

Across all focus groups, the majority of providers described physical activity as engaging in organized activities, such as throwing a ball, swinging, dancing, and climbing, or as general activity throughout their day, such as running around during free-play. The general consensus was that physical activity is an important part of children's daily routine at FCCHs. Although most providers reported children being very active throughout the day and agreed on the importance of physical activity as part of children's daily routine at their FCCHs, the amount of time providers believed children should engage in physical activity ranged from 30 minutes to two hours.

#### 3.3.2. Practices Related to Physical Activity

When asked to discuss how they ensure that children attending their FCCHs are physically active, the majority of providers described creative methods such as use of small outdoor and indoor equipment including hula hoops, jump rope, small trampolines for keeping children active throughout the year including during the cold winter months. Several providers described creative methods for keeping children active during cold winter months including having children use indoor equipment such as hula hoops, jump rope, and small trampolines.* “When we are inside, we use dancing a lot.” “I use the second floor staircase.”* Some providers spoke of making modifications to their homes, both indoors and outdoors to make it more conducive to physical activity.* “In my house we redid the basement, so the kids have a big space to play, jump, and use hula hoops.”*

Most providers felt that screen time should be regulated and that children should be allowed a maximum of one hour of screen time per day. TV viewing was the most common type of screen time. Many providers reported allowing children to have screen time during transition times such as dropoff, pickup, and meal preparation.* “I let them play computer games for about 15 minutes. And some TV when I am preparing the food, but no more than an hour a day.”* A few providers seemed to make a distinction between screen time for educational purposes and screen time for entertainment and felt that as long as the TV was being used for educational purposes it was “Ok” to let children watch 30–60 minutes of TV a day.* “I think it's Ok for the kids to watch some educational program on TV such as the PBS programs. Some of those programs are very good and teach the kids basic language skills.”* Although most providers reported that the majority of parents do not mind their kids watch some TV while at the FCCH, a few mentioned that they respect and find alternative for children whose parents do not want them to watch any TV while at FCCH.* “I have a parent who really does not want her daughter to watch TV while at daycare. I respect that, so when the other kids are watching TV, I have her draw.”* Although the majority of providers did not express concerns or challenges with limiting screen time, a few providers noted that some parents allowed their children to bring electronics to daycare even when there were rules against that.* “I have a hard time when parents don't respect the rules that I have around children bringing and using electronics such as DS. I don't really like to have to keep reminding them that those devices are not allowed in my FCCH.”* Nevertheless, a few providers noted that some parents allowed their children to bring electronics to daycare, even when there are rules against that, which was viewed as challenging.

#### 3.3.3. Barriers to Physical Activity

Across all focus groups, nearly all providers noted obstacles to children being physically active with lack of space and the cold weather being the most frequently noted.* “It's really hard to keep the kids active when it's cold and they don't have as much space to move around inside the house.”* In order to overcome these barriers some providers mentioned creative ways to ensure that children engage in PA while at their FCCHs.* “My home is not that big, so we often play a game and use the stairs. I have the kids go up and down a few times…” “My house is small, but I have a house with two garages and inside there is a ball, and things saved for winter, and we begin to throw a ball and move around in there and we maintain our activity level.”* Furthermore, some providers reported taking children to nearby parks or going for short walks around their homes, when the weather allowed.* “Whenever the weather is good I take the kids to a park near the house.”*

Many providers felt children were less active during the winter in comparison to the summer due to the cold weather when children spent more time indoors. A few providers noted that it can take a long time to get all children's winter gear on and off, and that can be especially challenging if they take care of several children. “*I only take them out once a day [in winter] because it is hard to put all the coats, gloves, hats.” *Furthermore, providers who had grown up outside the US, in warmer climates appeared to perceive the cold weather as a barrier more than providers who had grown up with cold weather.* “I was born in the Dominican Republic. I am not used to the kind of cold weather we get here in MA. I do not think I will ever get used to it … I just try to get through the winter. It can be difficult.”*

### 3.4. Communication with Parents 

#### 3.4.1. Attitudes and Practices Related to Communication with Parents

Nearly all providers spoke of the importance of having effective, open, and ongoing communication with parents as most children spend most of the day in their care. Most providers reported sharing information regularly with parents about children routines, what and how much they ate, and anything unusual such as an illness or injury. Furthermore, many providers felt that ongoing communication with parents is critical, as it allows them to understand children's home environment including family's routines and practices, how these shape the socio-emotional and physical development of the children they care for.* “Communication with parents is also very important because it gives us a chance to learn about the child's home environment, the family's routines and rules, which is really important information to have to understand and care for the child in our FCCHs.”*

#### 3.4.2. Communication Methods

Providers used multiple communication channels, including notes sent home at the end of day, forms, in-person communication, emails, texts, phone calls, and bulletin boards, where parents can see when they pick up/drop off their children. In fact, bulletin boards were used at most FCCHS.* “I tell them what the children have done, we talk about the progress of the child, what vocabulary they have learned, things like this, if the child has a necessity, how we can help the child, the resources in the community to help…”*

#### 3.4.3. Food-Related Communications

Several providers report sharing menu information with parents.* “I like to give the weekly menu to parents at the beginning of the week so that they understand and know what I am feeding their child at my FCCH and that I will serve a variety of foods*.*”* Other providers reported giving hard copies of a weekly/monthly meal menu to parents, while others reported posting it on an online bulletin board. A few providers reported using a software program provided by their local food program to report children's daily food consumption and physical activity. Providers who used such software stated they really liked using the software for its easiness of communication with parents.* “I like it because all the information can be easily emailed to parents*.*”*

#### 3.4.4. Communication Related to Weight Concerns

When asked how comfortable and confident they feel about talking with parents about any concerns they might have about a child' weight status, most providers reported feeling very comfortable and confident. However, the majority of providers reported that they did not have major concerns about weight status of children currently under their care. A couple of providers who reported having an overweight child under their care in the past mentioned that they felt comfortable and confident approaching the child's mother, discussing their concerns and sharing resources with the mother.* “A year or so ago, I had a child in my home (FCCH) who was overweight. So, I approached the mother and talked to her about my concern. I told her that I believed she should check with her pediatrician. I also told her about a training that I had attended and how during this training the instructor had stressed the importance of keeping children active, not allowing them to drink lots of sugar sweetened drinks, and having them eat plenty of fruits and vegetables…”*

A few providers stated that they did not feel comfortable discussing children's weight status with parents.* “It's kind of difficult because when I think of children I've encountered who were overweight, usually the parents are overweight.” *Providers who reported being reluctant to discuss child's weight felt that parents can be very sensitive to other people's perceptions of their children, and because of that they preferred not to talk about it with parents. Additionally, some providers felt that it is hard to change families' habits and that parents are not always open to advice.* “It is very difficult to educate the parent sometimes. Parents feed her a lot of McDonalds so I have tried to give them information on my menus so they can take [sic] home.” *There were, however, a few providers who felt it was their responsibility and part of their “job” to approach parents if they had concerns about a child's weight status. “*I feel it is part of my job to let parents know any concerns I have about their child, and that also goes for any concerns related to a child's weight status. I agree that it is not always easy to talk about it, and that parents can be sensitive sometimes, but I still think it is my responsibility.”*

## 4. Discussion

In this study, theory-driven qualitative research methods were used to assess Latino providers' beliefs and practices related to promoting healthful dietary and physical activity behaviors among preschool children attending FCCH. Theory-driven qualitative approaches are critical to enhancing knowledge and guiding development of interventions that promote healthful behaviors related to pediatric obesity intervention [22, 23].

Study findings indicate that Latino FCCH providers are vested in and believe they are influential in promoting healthy eating and physical activity behaviors of the preschool children in their care. In agreement with previous studies focusing on child care centers and family child care homes, Latino FCCH providers participating in our study perceived their role beyond simply “watching children” to one that includes promotion of early healthy behaviors including nutrition and physical activity [13, 24, 25].

Analysis revealed a few barriers and challenges faced by providers in establishing and maintaining healthful nutrition and physical activity practices in their FCCHs, including financial constraints. Several providers referred to high costs of fruit and vegetables, especially organic types, as a potential limiting factor. This finding is consistent with studies showing that low socioeconomic and neighborhood settings are an important factor influencing residents' consumption of healthy food choices, such as fruits and vegetables [14, 26].

Latino FCCH providers participating in our study reported using strategies such as, encouragement and role modeling to influence healthy food choice and consumption, particularly with regard to introducing new foods and increasing variety. Our findings are in agreement with that of a previous study by Hughes et al. [27] conducted with Latino Head Start providers highlighting the important influence that child care providers have in the development of healthy and unhealthy eating behaviors in minority children [27–29]. Contrary to a recent longitudinal survey study by Lanigan [30], Latino providers in our study did not report negative practices including pressuring child to eat and rewards for eating foods, although our qualitative study has a small sample size (*n* = 44).

Although most providers were consistent regarding mealtime food choices and routines, we found varied interpretations of portion size. This suggests that providers may benefit from additional training that assesses and addresses provider's knowledge and educates providers about evidence-based practices related to healthful eating and child feeding practices [31–33].

In general, Latino providers in our study perceived parents as not being aware of the importance of healthful eating practices and/or lacking the time needed to ensure that their children ate a healthful diet. In addition, most providers felt that it was part of their “job” to engage with and educate parents about the importance of proper child nutrition and healthy eating behaviors. This finding is consistent with findings from recent studies [13, 24, 34] which indicate the important role that child care providers can play in the promotion of children's early healthy behaviors related to eating and physical activity [13].

Our findings regarding providers' positive beliefs related to child nutrition and feeding practices suggest that regulations and resources, particularly those promulgated by CACFP, are important factors influencing Latino FCCH providers' knowledge and practices related to nutrition and child feeding. Providers spoke positively about educational opportunities available to them through training and workshops required for licensing of their FCCHs. This finding is in agreement with that of Stan et al. [31] documenting that broad-scale, in-person training is well received by child care providers and can be effective in increasing child care providers' knowledge of regulations to promote healthful eating and child feeding practices in child care settings, including FCCHs [32].

Our results revealed that in general, FCCH providers perceive physical activity as important for children's overall health. Nevertheless, we found that Latino FCCH providers in our study appear to have a wide range of concepts of what constitutes physical activity practices for children and reported a range of time in which they regularly implement physical for children in their FCCHs. These findings are consistent with those of previous studies [35, 36] showing a wide range of perceptions, knowledge, and practices related to physical activity among young children. Previous studies [37–39] have documented that caregivers' modeling of physical activity is influential in children's physical activity levels. Providers in our focus groups did not mention the importance of caregivers' physical activity level and modeling; therefore future training resources for promoting physical activity practices in FCCHs should highlight importance of caregiver physical activity level and modeling.

Our findings suggest less variation in providers' beliefs and practices related to screen time. Nearly all providers participating in our study reported believing that children should not be allowed more than one hour of screen time daily. Providers in our study spoke of using screen time (mostly educational TV programs) only during transition times (e.g., at pick up, preparation of lunch, etc.). Previous studies conducted among low-income population have shown high levels of TV-watching by children and adults [40, 41]. It is likely that providers participating in our study have been exposed to education and training and required to comply with regulatory policies that encourage limited use of TV and other screen devices set forth by agencies working with licensed FCCHs.

In agreement with previous studies, our findings revealed barriers to physical activity for children in FCCHs [35, 36] including cold weather and the physical environment of the FCCH that may lack appropriate indoor and outdoor spaces. Many providers in our study live in neighborhoods with small or no yard areas. Some reported financial constraints as limiting configuring indoor space for active play. This finding is consistent with previous studies, including our own [14, 41] with Latino parents, which found housing and neighborhood barriers faced by families living in low-income areas, with limited access to indoor and safe outdoor spaces [14, 34, 40, 41]. This is an important finding, and as suggested by previous studies [35, 36], physical activity interventions targeting family child care homes must be tailored to meet the unique characteristics of this home-based child care environment.

Specific cultural influences related to Latino FCCH providers' beliefs, attitudes, and practices related to nutrition and physical activity were not widely apparent in our study. Although some providers reported serving foods typical to the Latino culture such as rice and beans on a regular basis, it is possible that other cultural influences reported by previous studies conducted among Latino populations such as, consumption of sugar-sweetened beverages and use of TV may be less present in licensed Latino FCCHs due regulatory agency requirements.

Finally, given the pivotal role that parents have in structuring home environment [12], it is important to note that the Latino FCCH providers in our study perceive that parental home environment is lacking in nutrition and physical activity structure. FCCH providers may be well positioned given their daily and close relationship with parents to engage and educate low-income, Latino parents about the importance of establishing a home environment conducive to the development of early healthy behaviors related to children's eating and physical activity. Our findings highlight the important influence and role that Latino FCCH providers can have as a unit of change and promotion of health in low-income, Latino communities. Interventions involving FCCH providers may prove to be an effective way to target low-income minority families for obesity prevention efforts. Given FCCH providers established presence in their communities, they are well positioned to facilitate low-income families' access to evidence-based information in a linguistic and culturally sensitive way. Latino providers have established trusting and respected relationships with Latino parents, which positions their family child care homes as an important venue for the delivery of long-term and sustainable efforts to prevent childhood obesity among at-risk, minority communities. The potential role of minority FCCH providers should be explored in future community-based interventions aimed at promoting healthful family behaviors related to nutrition and physical activity.

Results of this study should be considered in light of some limitations. Findings are based on a nonrandom, purposive, and relatively small sample of low-income, Latino FCCH providers in four selected communities in Massachusetts. Furthermore, FCCH providers recruited to participate in this study could have been those who are more aware and concerned in general with promoting health behaviors among children in their care. Future research can address these limitations by exploring influences on Latino providers' beliefs, attitudes, and practices from other communities across the US. In addition, quantitative research that builds on the qualitative findings reported here is needed to quantify Latino providers' beliefs, attitudes, and practices related to the promotion of healthy eating and physical activity behaviors among Latino preschool children attending FCCHs.
